# Circadian Phase Shapes Muscle-Derived Extracellular Vesicle microRNA Profiles with Context-Dependent Modulation by Exercise in High-Fat-Diet-Fed Mice

**DOI:** 10.3390/nu18071076

**Published:** 2026-03-27

**Authors:** Shuo Wang, Noriaki Kawanishi, Cong Wu, Haruki Kobori, Katsuhiko Suzuki

**Affiliations:** 1 Graduate School of Sport Sciences, Waseda University, Tokorozawa 359-1192, Japan; 2 Faculty of Advanced Engineering, Chiba Institute of Technology, Chiba 275-0023, Japan; 3 Faculty of Sport Sciences, Waseda University, Tokorozawa 359-1192, Japan

**Keywords:** extracellular vesicles, microRNA, circadian rhythm, exercise, skeletal muscle, high-fat diet

## Abstract

**Background:** Extracellular vesicles (EVs) released from skeletal muscle mediate metabolic communication via microRNAs (miRNAs). While both circadian rhythms and exercise influence metabolism, the joint modulation of the muscle-derived EV miRNA landscape by circadian rhythms and chronic exercise remains undefined, particularly under the metabolic stress of obesity. **Methods:** Employing a 2 × 2 factorial design (Phase: ZT3 vs. ZT15; Condition: sedentary vs. exercise; ZT, Zeitgeber Time), EV-enriched fractions were isolated from ex vivo quadriceps muscle (QUA) cultures of high-fat diet-fed mice following an 8-week treadmill training regimen using polymer-based precipitation, and comprehensive miRNA profiling was performed by small RNA sequencing. **Results:** Principal component analysis (PCA) revealed that circadian phase accounted for a greater proportion of global variance in EV miRNA profiles than exercise. Differential expression analysis identified miR-1a-3p and miR-1b-5p as upregulated across both composite phase and exercise contrasts; however, condition-specific analyses indicated that this signal was primarily driven by the sedentary-phase comparison (ZT15-sed vs. ZT3-sed), in which the miR-29 family was also prominently co-upregulated, rather than constituting independent phase and exercise effects; this phase-associated signature was absent in the corresponding exercise-condition comparison. Exploratory functional enrichment of experimentally validated targets revealed phase-preferential association with metabolic and iron–heme pathways, whereas exercise-associated miRNAs mapped to signaling, inflammatory, and transcription-related networks. **Conclusions:** Circadian phase was the dominant contributor to global variance in muscle-derived EV-enriched miRNA profiles in obesity, as reflected by the phase-associated separation along principal component 1 (PC1, 33.47% of total variance), with exercise introducing context-dependent adaptive modulation. This study provides a foundational basis for investigating the temporal regulation of muscle secretome dynamics under high-fat diet conditions, highlighting temporal specificity as a key dimension in EV-mediated exercise physiology research.

## 1. Introduction

Extracellular vesicles (EVs) are membrane-enclosed particles released by most cell types. Small EVs, often operationally defined as vesicles < 200 nm in diameter, represent one subclass of EVs. They carry diverse molecular cargoes, including proteins, lipids, messenger RNAs (mRNAs), and microRNAs (miRNAs). Through cargo delivery, EVs participate in intercellular and interorgan communication, as summarized in current EV consensus guidelines [1].

Skeletal muscle is a major contributor to metabolic homeostasis and physical activity [2]. It communicates with distal organs through the secretion of myokines and muscle-derived EVs [3]. Accumulating evidence suggests that acute endurance exercise triggers a rapid systemic release of small EVs, whose miRNA and protein profiles are transferred to recipient tissues, such as the liver, to mediate adaptive metabolic signaling [3,4]. However, chronic metabolic stress induced by a high-fat diet (HFD) disrupts this EV-mediated signaling network: skeletal muscle-derived EVs exhibit alterations in both secretion volume and molecular heterogeneity, particularly in their lipid and miRNA composition, which may contribute to muscle homeostatic imbalance [5]. Furthermore, EVs released from lipid-induced insulin-resistant muscle cells can alter gene expression and proliferation in pancreatic β-cells [6], leading to a vicious cycle of insulin resistance.

miRNAs are 21–25-nucleotide non-coding ribonucleic acids (RNAs) that regulate gene expression by binding to the 3′ untranslated regions of target mRNAs [7]. EV-encapsulated miRNAs are protected by a lipid bilayer, contributing to their relative stability in extracellular environments [1]. Physical exercise has been shown to modify the composition of circulating EV miRNAs, such as miR-133a, miR-181, and the let-7 family [3], though the correspondence between muscle and circulating exosomal miRNA abundances following acute exercise remains inconsistent [8]. However, most prior research has focused on the transient effects of acute exercise. The extent to which long-term training and, crucially, the timing of exercise influence the chronic adaptation of the muscle-derived EV miRNA profile remains an unresolved question in metabolic physiology [3,4,8].

The circadian clock exerts time-dependent regulation on nearly all physiological processes [9], with over 40% of mammalian genes exhibiting circadian rhythmicity [10]. Skeletal muscle acts as a peripheral clock where gene expression and metabolism are governed by core clock proteins [11]. Notably, the metabolic efficacy of exercise is highly phase-dependent: exercise performed during the active phase improves energy metabolism more effectively than rest-phase interventions [12], paralleling the diurnal peaks in muscle performance [13]. Despite these insights, the circadian orchestration of EV secretion and EV miRNA composition remains poorly understood. While circadian rhythmicity has been demonstrated at the transcriptome level [10], specific miRNAs have also been reported to exhibit circadian oscillation in peripheral tissues [14]. Whether this translates to temporal variation in EV miRNA cargo is unexplored. Moreover, under HFD conditions, where metabolic homeostasis is disrupted, how circadian timing and chronic exercise jointly modulate EV-mediated interorgan communication remains uncharacterized. Given that EVs serve as key mediators of metabolic crosstalk between muscle and peripheral tissues, elucidating their circadian dynamics could reveal novel mechanisms underlying exercise’s time-dependent metabolic benefits [3,5].

This study was designed as a discovery-driven investigation to develop hypotheses regarding the joint modulation of muscle-derived EV-enriched miRNA profiles by circadian phase and chronic exercise under HFD conditions. Using a 2 × 2 factorial design (Phase: ZT3 vs. ZT15; Condition: sed vs. exe; ZT, Zeitgeber Time; sed, sedentary; exe, exercise), this study examines (i) phase- and exercise-dependent changes in particle size characteristics of EV-enriched preparations from ex vivo quadriceps muscle (QUA) cultures; (ii) phase- and exercise-associated differences in EV miRNA expression; and (iii) experimentally validated target-gene networks and functional enrichment of key differentially expressed miRNAs. The results are intended to provide a foundational basis for chronobiological investigation of muscle-derived EV miRNA signaling under obesogenic conditions.

## 2. Materials and Methods

### 2.1. Experimental Animals and Circadian Phase Manipulation

All experimental procedures were approved by the Animal Experiment Review Committee of Waseda University (approval number: A23-126) and conducted in accordance with institutional guidelines and the Animal Research: Reporting of In Vivo Experiments (ARRIVE) 2.0 guidelines. Seven-week-old healthy male C57BL/6J mice were obtained from Takasugi Experimental Animals Supply Co., Ltd. (Kasukabe, Japan). Animals were housed in plastic cages (2–3 per cage) at 22 ± 2 °C under a 12:12 h light–dark cycle (lights on at 08:00 and off at 20:00), with ad libitum access to water and HFD (Research Diets D12492, E.P. Trading Co., Ltd., Tokyo, Japan; protein 20 kcal%, carbohydrate 20 kcal%, and fat 60 kcal%).

The overall experimental design, including circadian manipulation and exercise protocols, followed our previous study [15]. Mice underwent 8 weeks of HFD feeding followed by 8 weeks of treadmill training or sedentary control in a two-factor (2 × 2) experimental design combining circadian phase (ZT3 vs. ZT15) and training condition (exercise vs. sedentary), with *n* = 6 mice per group. ZT0 was defined as lights on; ZT3 corresponded to 3 h after lights on (rest phase) and ZT15 to 3 h after lights off (active phase). ZT3 and ZT15 were selected to represent the early rest and early active phases of nocturnal mice, respectively, based on established phase-dependent exercise responses [12,13] and consistent with our previous study [15]. Mice assigned to the ZT15 group were exposed to 24 h of constant light on Day 3 of HFD feeding, followed by a 12 h light–dark reversal (new ZT0 = 20:00; lights on at 20:00, lights off at 08:00). This reversed light–dark schedule was maintained for the remainder of the experiment, ensuring that both phase groups trained at the same local clock time (11:00) under otherwise identical environmental conditions. This procedure produced a stable 12 h phase delay relative to the ZT3 group while preserving the 24 h rhythm [16,17]. Re-entrainment of the ZT15 group was confirmed in our previous study [15] by monitoring the active/rest feeding ratio, which stabilized within approximately 12 days after the light–dark reversal, confirming successful phase separation prior to the exercise intervention.

### 2.2. Exercise Intervention and Tissue Collection

To minimize baseline body weight differences, animals were initially allocated using pairwise randomization based on body weight. After the HFD period, each phase group was subdivided into sedentary and exercise subgroups using the same pairwise randomization method based on body weight reassessed at Week 8. Exercise training was conducted 5 consecutive days per week, with 2 days of rest on weekends, for 8 weeks at 60 min per session at either ZT3 or ZT15, following a progressive moderate-to-vigorous aerobic protocol targeting approximately 60–70% of maximal oxygen uptake (VO_2_max) for obese C57BL/6J mice [18,19]. Treadmill speed began at 9 m/min in Week 9 (i.e., the first week of training), increasing by 2 m/min every 10 min during the first 20 min, after which speed was held constant for the remainder of each session; the starting speed was advanced by 1 m/min per week, reaching 13 m/min by Week 13, at which speed it was maintained for the remainder of the training period. Sedentary controls were placed near the treadmill during training hours but did not perform running. Forty-eight hours after the final exercise session, mice were fasted for 4 h and euthanized for QUA collection to minimize acute exercise effects and feeding effects. No animals or samples were excluded from the analysis. No formal a priori sample size calculation was performed; group sizes were determined based on feasibility and precedent from prior studies. Group allocation was known to the investigators, and no blinding was applied at any stage of the experiment.

### 2.3. Ex Vivo Culture of QUA Tissue and EV Isolation

To collect muscle-derived EV-enriched conditioned media, ex vivo explant cultures of QUA tissue were established as follows [20,21]: QUA tissue was rinsed three times with phosphate-buffered saline (PBS), cut into approximately 5 mm fragments, and incubated in 6-well plates containing 6 mL of serum-free Dulbecco’s Modified Eagle Medium (DMEM) supplemented with 1% penicillin–streptomycin. Cultures were maintained at 37 °C under 5% CO_2_ for 24 h. The resulting conditioned media were then collected for EV isolation, particle size characterization, and EV-associated miRNA profiling. Conditioned media were centrifuged (3000× *g*, 15 min) to remove cellular debris, followed by filtration through a 0.22-μm filter. EVs were enriched using the Total Exosome/EV Isolation Reagent (Thermo Fisher Scientific, Waltham, MA, USA) following the manufacturer’s instructions. As precipitation-based methods may co-isolate non-vesicular components, the preparations are described as EV-enriched in accordance with Minimal Information for Studies of EVs (MISEV) 2018 guidelines [1], and the results should be interpreted as reflecting the miRNA landscape of the muscle-derived EV-enriched secretome.

### 2.4. EV Size Distribution

Particle size was measured by nanoparticle tracking analysis (NTA) using the VideoDrop system (Meiwafosis Co., Ltd., Tokyo, Japan). For EV size analysis, we analyzed *n* = 6 independent biological replicates per group, with each replicate representing one mouse. Each sample was serially diluted (1:10–1:1000) to ensure that particle counts fell within the linear detection range. For each sample, three videos were recorded at 25 °C under identical tracking settings. Particle size distributions were binned at 50 nm intervals and normalized to total counts. Size metrics (mean and median diameters) are presented as descriptive summaries: mean ± standard error of the mean (SEM) for mean diameter and box-and-whisker plots for median diameter.

### 2.5. Small RNA Extraction, Library Preparation, and Sequencing

For miRNA sequencing, to obtain sufficient RNA yield for library preparation, the six mice in each experimental group were randomly assigned into three independent pairs, and EV samples from each pair of mice were pooled to form one biological replicate. Consequently, each of the four experimental groups (ZT3-sed, ZT3-exe, ZT15-sed, and ZT15-exe) comprised *n* = 3 independent biological replicates for sequencing, with each replicate derived from a pool of two mice. Total RNA was extracted from EV-enriched fractions using the Total Exosome RNA & Protein Isolation Kit (Thermo Fisher Scientific, Waltham, MA, USA). The concentration of the extracted small RNA was quantified using a Qubit^®^ 3 Fluorometer with the Qubit RNA High Sensitivity Assay Kit (Thermo Fisher Scientific). Given that the target analytes were small RNAs (<200 nt), formal assessment of the RNA Integrity Number via Bioanalyzer was not performed. Libraries were generated with the QIAseq^®^ miRNA Library Kit and QIAseq^®^ miRNA next-generation sequencing (NGS) Index Kit (Qiagen, Hilden, Germany), including adaptor ligation, reverse transcription, and amplification with 22 polymerase chain reaction (PCR) cycles. Library concentrations were quantified using a Qubit^®^ 3 Fluorometer and normalized to equimolar amounts. Sequencing was performed on the Ion GeneStudio™ S5 system (Thermo Fisher Scientific) with approximately 100 bp single-end reads, yielding more than 6 million reads per library.

Sequencing reads were processed via the Qiagen GeneGlobe Data Analysis Center and mapped to miRBase Release 22.1 [22]. miRNAs with a total read count greater than 10 were retained for analysis [23].

### 2.6. Differential Expression Analysis of miRNAs

Raw count data were analyzed in DESeq2 (Galaxy v2.11.40.8) [24] with median-of-ratios normalization. Given the limited sample size (*n* = 3 biological replicates per group), statistical power for reliably detecting interaction effects in a full factorial model would be insufficient; differential expression was therefore examined using a set of predefined contrasts designed to describe phase- and exercise-related expression differences across groups, without an explicit interaction term. For robust inference, differential expression tests prioritized miRNAs with BaseMean ≥ 25. The analysis comprised two types of contrasts:

(i) Composite-effect contrasts to assess overall trends: all ZT15 vs. all ZT3 samples (composite phase contrast); all exe vs. all sed samples (composite exercise contrast).

(ii) Condition-specific pairwise comparisons to resolve context-dependence: ZT15-sed vs. ZT3-sed; ZT15-exe vs. ZT3-exe; ZT3-exe vs. ZT3-sed; and ZT15-exe vs. ZT15-sed.

Each contrast was tested independently using the Wald test. For exploratory visualization (volcano plots), miRNAs meeting the criteria of BaseMean ≥ 25, an absolute log_2_ fold change (log_2_FC) > 0.30, and *p* < 0.05 were highlighted. For downstream intersection analysis of target gene sets (Section 2.8) and functional enrichment analysis (Section 2.9), miRNA sets were constructed from all six contrasts listed above using a more permissive exploratory threshold (BaseMean ≥ 25, an absolute log_2_FC > 0.30, and *p* < 0.10). This expanded set was used exclusively for hypothesis generation.

### 2.7. Principal Component Analysis (PCA)

PCA was performed to evaluate the global variation structure of EV miRNA expression profiles. The analysis was based on variance-stabilizing transformation (VST) data generated by DESeq2 on the Galaxy platform [24,25], followed by mean-centering after data export. PCA visualization and result reporting were conducted using GraphPad Prism 10 (GraphPad Software, San Diego, CA, USA).

The PCA included all detected miRNAs (155 miRNAs) across all samples (*n* = 12; four experimental groups with *n* = 3 biological replicates per group, each replicate representing an independent pool derived from two mice). No differential-expression filtering was applied to capture overall miRNA expression structure rather than differential effects. Eigenvalues, percentage variance explained, and cumulative variance contributions are reported for the first two principal components (PC1 and PC2).

### 2.8. Intersection Analysis of Target-Gene Sets Derived from Composite and Condition-Specific Comparisons

To assess the consistency between composite effects (Phase or Exercise) and condition-specific miRNA patterns, target gene sets were constructed based on differentially expressed miRNAs identified using the exploratory threshold described in Section 2.6 (BaseMean ≥ 25, an absolute log_2_FC > 0.30, *p* < 0.10). Target gene information was obtained exclusively from experimentally validated miRNA–target interactions curated in the miRNA Target Interaction Database (miRTarBase) Release 10. miRNAs without experimentally validated target information were excluded from downstream analyses. Prediction-based databases were not used.

Differentially expressed miRNAs were first categorized into upregulated and downregulated sets for the composite phase effect and the composite exercise effect, respectively. For each composite effect, only the two corresponding condition-specific comparisons were considered (e.g., for the composite phase effect, ZT15-sed vs. ZT3-sed and ZT15-exe vs. ZT3-exe), and upregulated and downregulated miRNA sets were constructed accordingly. Based on these miRNA sets, target gene sets were generated for both the composite effects and their corresponding condition-specific comparisons. Intersections among these gene sets were examined to identify core target genes, defined as genes that belonged to a composite-effect target gene set (Phase or Exercise) and were also present in at least one of the two corresponding condition-specific comparison target gene sets. These intersections were visualized using Venn diagrams generated with the jvenn online tool [26].

### 2.9. Functional Enrichment Analysis of Target Genes

Core target genes, defined in Section 2.8, were used for downstream functional enrichment analyses, performed separately for upregulated and downregulated sets under phase and exercise effects.

Gene Ontology biological process (GO-BP) enrichment (GOTERM_BP_DIRECT) and Kyoto Encyclopedia of Genes and Genomes (KEGG) pathway enrichment analyses were performed using Database for Annotation, Visualization and Integrated Discovery (DAVID) 2025 (v2025_1) [27,28,29], with Mus musculus as the reference background in DAVID and official gene symbols used as identifiers. Multiple testing correction was applied using the Benjamini–Hochberg procedure. For GO-BP enrichment, terms with a Benjamini–Hochberg-adjusted false discovery rate (FDR) < 0.05 and a gene count (Count) ≥ 5 were considered statistically significant. For KEGG pathway enrichment of upregulated target genes, the same significance criteria were applied (FDR < 0.05, Count ≥ 3). For KEGG pathway enrichment of downregulated target genes, a more permissive threshold was used (FDR < 0.10, Count ≥ 3) to avoid excessive exclusion of potentially relevant pathways when pathway representation was limited.

Functional enrichment analyses were positioned as exploratory, and the results were interpreted primarily for hypothesis generation rather than for defining definitive pathway activation. For result presentation, representative GO-BP terms and KEGG pathways were selected from the significantly enriched results based on their physiological relevance to the focus of this study.

### 2.10. Statistical Analysis

Particle size distributions were normalized to total counts and are presented descriptively as mean ± SEM (mean diameter) or box-and-whisker plots (median diameter). For small RNA sequencing, each experimental group comprised *n* = 3 biological replicates (pooled pairs of mice). Raw count data were analyzed using DESeq2 (Galaxy v2.11.40.8) with median-of-ratio normalization. Differential expression was evaluated using predefined composite and condition-specific contrasts without inclusion of an interaction term due to limited statistical power. Wald tests were applied independently for each contrast. For exploratory visualization (volcano plots), miRNAs meeting BaseMean ≥ 25, absolute log_2_FC > 0.30, and *p* < 0.05 were highlighted. For target gene intersection analyses, a more permissive exploratory threshold (BaseMean ≥ 25, absolute log_2_FC > 0.30, *p* < 0.10) was applied for hypothesis generation. PCA was performed on VST data, including all detected miRNAs (*n* = 155; *n* = 12 samples) without prior filtering. Functional enrichment analyses were performed as described in Section 2.9 and interpreted as exploratory. All analyses were conducted in an exploratory framework without a prespecified primary outcome measure.

## 3. Results

### 3.1. Size Characteristics of EVs in HFD Mice

**Figure 1:**
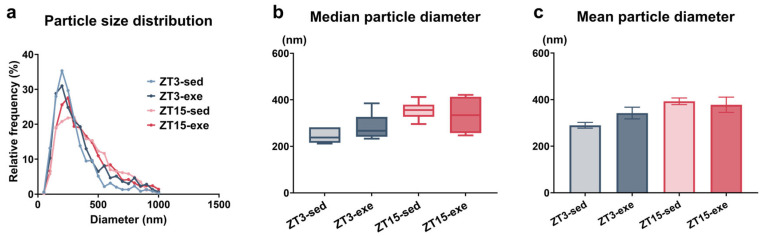
Size characteristics of QUA-derived EV-enriched particles: (**a**) Size distribution curves (50 nm bins; normalized to total counts). (**b**) Median diameter (box = 25–75%; whiskers = 10–90%; *n* = 6). (**c**) Mean diameter (mean ± SEM, *n* = 6 per group). Data were obtained by NTA. QUA, quadriceps muscle; EV, extracellular vesicle; SEM, standard error of the mean; NTA, nanoparticle tracking analysis; ZT, Zeitgeber Time; sed, sedentary; exe, exercise.

To describe the particle size characteristics of EV-enriched preparations for downstream miRNA profiling, we profiled the particle size of ex vivo QUA-derived EV-enriched preparations using NTA (Figure 1). Size distributions were unimodal with a predominant peak between 100 and 400 nm (Figure 1a). Descriptively, median and mean diameters were numerically larger in ZT15 groups than ZT3 groups, whereas no clear directional pattern was observed between exercise and sedentary conditions (Figure 1b,c). Given the polymer-based isolation method and potential particle aggregation, these size metrics are interpreted as preparation characteristics rather than evidence of differential vesicle biogenesis.

### 3.2. Composite Trends and Condition-Specific Patterns in EV miRNA Expression

**Figure 2:**
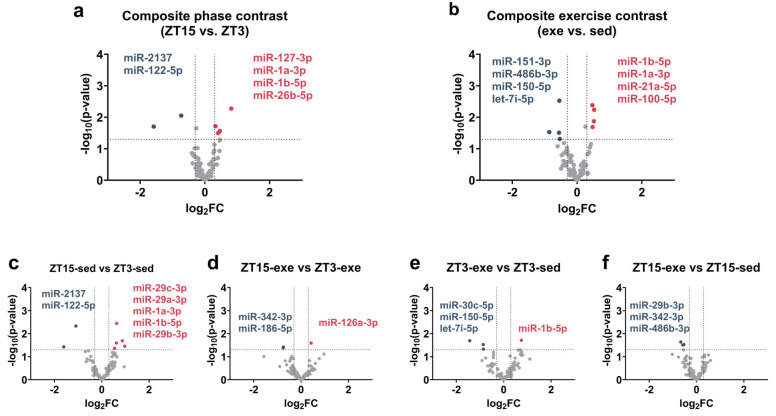
Volcano plots of differential miRNA expression. Panels (**a**,**b**) show composite contrasts for Phase and Exercise, respectively. Panels (**c**–**f**) show pairwise (condition-specific) comparisons: (**c**) ZT15-sed vs. ZT3-sed, (**d**) ZT15-exe vs. ZT3-exe, (**e**) ZT3-exe vs. ZT3-sed, and (**f**) ZT15-exe vs. ZT15-sed. Exploratory thresholds were set at BaseMean ≥ 25, an absolute log_2_FC > 0.30, and *p* < 0.05. Axes represent log_2_FC (*x*-axis) and −log_10_(*p*-value) (*y*-axis). Dashed lines indicate cutoff values. Colors denote regulation status: red, upregulated; blue, downregulated; gray, non-significant. Sample size = 3 biological replicates per group (pooled pairs of mice). Differential expression analysis was performed using DESeq2 (Wald test). miRNA, microRNA; FC, fold change.

To summarize overall expression trends, we first examined the composite effects of phase and exercise by comparing pooled sample groups (Figure 2). Using the exploratory thresholds described in Methods, Section 2.6 (BaseMean ≥ 25, an absolute log_2_FC > 0.30, *p* < 0.05), six miRNAs met the criteria in the composite phase contrast (ZT15 vs. ZT3) (Figure 2a): four upregulated (miR-127-3p, miR-1a-3p, miR-1b-5p, and miR-26b-5p) and two downregulated (miR-2137 and miR-122-5p). For the composite exercise contrast (exe vs. sed), eight miRNAs met these criteria (Figure 2b): four were upregulated (miR-1b-5p, miR-1a-3p, miR-21a-5p, and miR-100-5p), and four were downregulated (miR-151-3p, miR-486b-3p, miR-150-5p, and let-7i-5p). Notably, miR-1a-3p (Phase contrast: log_2_FC = 0.46, *p* = 0.027; Exercise contrast: log_2_FC = 0.52, *p* = 0.013) and miR-1b-5p (Phase contrast: log_2_FC = 0.41, *p* = 0.032; Exercise contrast: log_2_FC = 0.53, *p* = 0.006) appeared in both composite contrasts. As the composite exercise contrast pools ZT3 and ZT15 samples together, and these miRNAs were prominently upregulated in the sedentary-phase comparison (ZT15-sed vs. ZT3-sed; Figure 2c) but not in the exercise-condition comparisons, their appearance in both composite contrasts reflects shared signal structure rather than two independent regulatory effects.

To dissect the context-dependent regulation, we performed four planned pairwise comparisons between specific experimental groups (Figure 2). Under sedentary conditions (ZT15-sed vs. ZT3-sed), seven miRNAs met the criteria, representing the largest set among the four contrasts; five were upregulated (miR-29c-3p, miR-29a-3p, miR-1a-3p, miR-1b-5p, and miR-29b-3p), and two were downregulated (miR-2137 and miR-122-5p) (Figure 2c). Under exercise conditions (ZT15-exe vs. ZT3-exe), three miRNAs met the criteria, including one upregulated miRNA (miR-126a-3p) and two downregulated miRNAs (miR-342-3p and miR-186-5p) (Figure 2d). For the exercise effect at ZT3 (ZT3-exe vs. ZT3-sed), four miRNAs met the criteria (one up: miR-1b-5p; three down: miR-30c-5p, miR-150-5p, and let-7i-5p) (Figure 2e). At ZT15 (ZT15-exe vs. ZT15-sed), three miRNAs were downregulated (miR-29b-3p, miR-342-3p, and miR-486b-3p) (Figure 2f). These results demonstrated that miRNA responses to phase and exercise were context-dependent across comparisons (complete statistics for all detected miRNAs across all six contrasts are provided in Table S1).

### 3.3. PCA Reveals a Dominant Phase-Associated Distributional Trend

**Figure 3:**
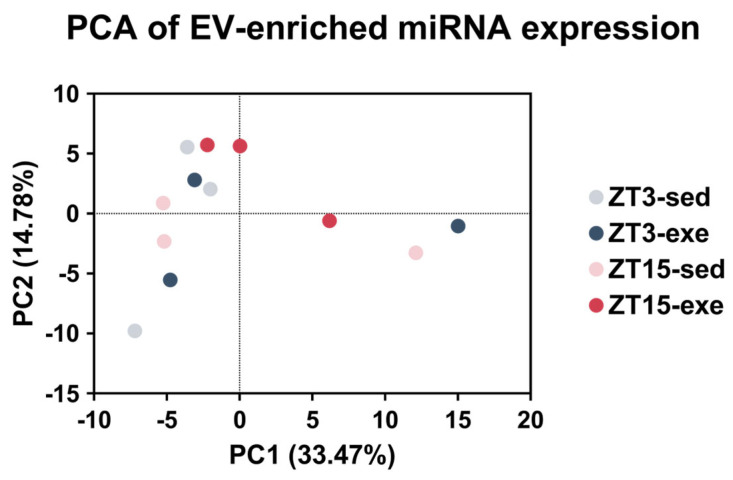
PCA of EV miRNA profiles. PCA of VST-transformed data (155 detected miRNAs) performed in GraphPad Prism. Each point represents one sample (*n* = 12; four groups with *n* = 3 biological replicates per group). PC1 explained 33.47% of the variance, and PC2 explained 14.78%, with a cumulative variance of 48.25%. Samples showed a phase-associated distribution along PC1 with partial overlap between groups. PC, principal component; PCA, principal component analysis; VST, variance-stabilizing transformation.

PCA was performed on the miRNA expression data (the VST-transformed miRNA expression matrix; 155 miRNAs across 12 samples). PC1 explained 33.47% of the total variance and showed a phase-associated separation: ZT3 samples distributed toward negative or near-zero PC1 values, whereas ZT15 samples distributed toward positive PC1 values, with partial overlap between groups (Figure 3). One ZT3-exe sample was also distributed toward positive PC1 values. PC2 accounted for 14.78% of the variance and captured additional inter-sample variability without separation by exercise condition. The cumulative variance explained by PC1 and PC2 was 48.25%. Overall, PCA demonstrated a greater contribution of circadian phase to global variance in EV miRNA expression, as shown by the phase-associated distribution along PC1.

### 3.4. Overlap Between Target Gene Sets from Composite and Condition-Specific Comparisons

**Figure 4:**
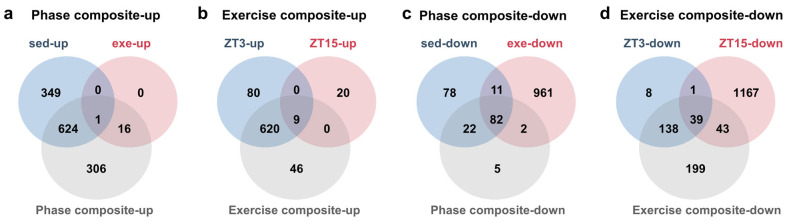
Intersections between composite-effect and corresponding condition-specific comparison target-gene sets. Venn diagrams illustrate the overlap between miRTarBase-validated target genes of miRNAs meeting the exploratory expansion criteria identified from the composite effects of phase or exercise and their corresponding condition-specific comparisons: (**a**) Intersections between phase composite-up targets and phase condition-specific comparison targets under sedentary (sed-up: ZT15-sed vs. ZT3-sed) and exercise (exe-up: ZT15-exe vs. ZT3-exe) conditions. (**b**) Intersections between exercise composite-up targets and exercise condition-specific comparison targets at ZT3-up (ZT3-exe vs. ZT3-sed) and ZT15-up (ZT15-exe vs. ZT15-sed). (**c**,**d**) The corresponding intersections for phase composite-down and exercise composite-down target sets, respectively. Numbers denote the number of genes in each region. Target genes were obtained from miRTarBase (Release 10; Mus musculus). Differential expression thresholds for exploratory intersection analysis were BaseMean ≥ 25, an absolute log_2_FC > 0.30, and *p* < 0.10. Venn diagrams were generated using jvenn. Sample size was *n* = 3 per group; each replicate was pooled from two mice (six mice per group total). miRTarBase, miRNA Target Interaction Database.

To examine the consistency between composite effect trends and condition-specific patterns, miRTarBase-validated target genes from all comparisons (Table S2) derived from miRNAs meeting an exploratory expansion threshold (BaseMean ≥ 25, an absolute log_2_FC > 0.30, *p* < 0.10) were intersected after gene-symbol deduplication (Figure 4). In the Phase composite-up group, 641 of 947 target genes (67.69%) overlapped with at least one condition-specific gene set (sed-up and/or exe-up), whereas 306 targets (32.31%) were unique (Figure 4a). In the Exercise composite-up group, 629 of 675 target genes (93.19%) overlapped with at least one condition-specific gene set (ZT3-up and/or ZT15-up), with only 46 targets (6.81%) unique to the Exercise composite-up group (Figure 4b). In the Phase composite-down group, 106 of 111 target genes (95.50%) overlapped with condition-specific gene sets, with only 5 targets (4.50%) unique (Figure 4c). By contrast, in the Exercise composite-down group, 220 of 419 target genes (52.51%) overlapped with condition-specific gene sets (ZT3-down and/or ZT15-down), while 199 targets (47.49%) were unique (Figure 4d).

These overlaps demonstrate that composite trends capture a substantial component of the condition-specific patterns; the unique sectors, particularly within exercise-related contrasts, confirm context-dependent regulation.

### 3.5. GO-BP Enrichment of Core Target Genes

To investigate the biological functions mediated by phase- and exercise-associated miRNAs, Gene Ontology (GO) enrichment analysis was performed on miRTarBase-validated target genes of core phase-up, exercise-up, phase-down, and exercise-down miRNAs, where “core” targets are those shared between a composite effect and at least one of its corresponding condition-specific comparisons. GO terms displayed were selected from the significantly enriched results based on physiological relevance to the focus of this study (complete GO-BP enrichment results for all core target gene sets are provided in Table S3).

**Figure 5:**
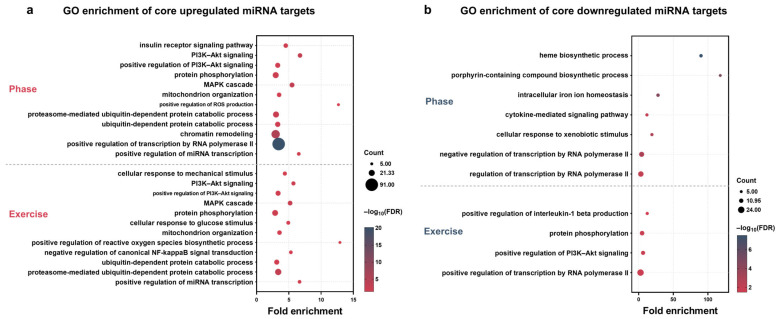
GO-BP enrichment analysis of core miRNA target genes. Bubble plots show GO enrichment of core upregulated miRNA target genes (**a**) and core downregulated miRNA target genes (**b**). In each panel, phase-related (Phase) core targets are shown at the top, and exercise-related (Exercise) core targets are shown at the bottom ((**a**): Phase, *n* = 616; Exercise, *n* = 605; (**b**): Phase, *n* = 105; Exercise, *n* = 213; *n* indicates the number of genes used for DAVID GO enrichment analysis). The *x*-axis represents fold enrichment. Dot size indicates the number of genes in each GO term (Count), and dot color represents the −log_10_(FDR). GO enrichment analysis was performed using DAVID 2025 (v2025_1) with Mus musculus used as the reference background in DAVID. Significance was defined as an FDR < 0.05 with Count ≥ 5. GO-BP, Gene Ontology biological process; GO, Gene Ontology; FDR, false discovery rate; DAVID, Database for Annotation, Visualization and Integrated Discovery; ROS, reactive oxygen species.

Target genes of core phase-up miRNAs were enriched in biological process terms related to metabolic and intracellular signaling processes, including insulin receptor signaling and phosphoinositide 3-kinase (PI3K)/protein kinase B (Akt) signal transduction. Additional enriched biological processes were related to protein phosphorylation, mitogen-activated protein kinase (MAPK) signaling, mitochondrial organization, and oxidative stress–related processes. The enrichment of these terms potentially reflects a temporal coordination of redox homeostasis across different circadian phases, complemented by the regulation of transcription and miRNA transcription (Figure 5a). Target genes of core exercise-up miRNAs showed enrichment in biological processes associated with mechanical and metabolic signaling. These included cellular response to mechanical stimuli, PI3K/Akt and MAPK signaling pathways, protein phosphorylation, mitochondrial organization, and regulation of miRNA transcription, together with oxidative stress-associated signaling and protein turnover (Figure 5a). For core phase-down miRNAs, enriched biological processes were predominantly related to heme biosynthesis and iron ion homeostasis. In addition, cytokine-mediated signaling and stress-related processes, as well as transcriptional repression by RNA polymerase II, were also enriched (Figure 5b). Target genes of core exercise-down miRNAs were enriched in inflammation-, transcription-, and signaling-related processes, including positive regulation of interleukin-1 beta (IL-1β) production, protein phosphorylation, and PI3K/Akt-related signaling (Figure 5b).

Collectively, these enrichment patterns suggest that phase- and exercise-associated miRNAs may map to partly distinct biological process modules, with phase-related core miRNAs (defined as those shared between composite and condition-specific comparisons) preferentially associated with metabolic and iron–heme-related pathways, and exercise-related miRNAs more strongly associated with signaling, inflammatory, and transcription-related processes.

### 3.6. KEGG Pathway Enrichment of Core Target Genes

To further characterize signaling pathways potentially mediated by phase- and exercise-associated miRNAs, KEGG pathway enrichment analysis was performed on miRTarBase-validated target genes of core phase-up, exercise-up, phase-down, and exercise-down miRNAs (as defined in Section 3.5). KEGG pathways were selected using the same criteria (complete KEGG pathway enrichment results for all core target gene sets are provided in Table S4).

**Figure 6:**
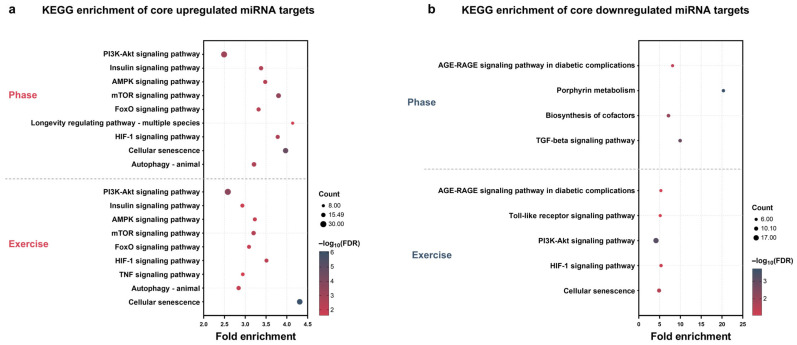
KEGG pathway enrichment analysis of core miRNA target genes. Bubble plots show KEGG pathway enrichment of core upregulated miRNA target genes (**a**) and core downregulated miRNA target genes (**b**). In each panel, phase-related (Phase) core targets are shown at the top, and exercise-related (Exercise) core targets are shown at the bottom ((**a**): Phase, *n* = 303; Exercise, *n* = 303; (**b**): Phase, *n* = 70; Exercise, *n* = 107; *n* indicates the number of genes used for DAVID KEGG enrichment analysis). The *x*-axis represents fold enrichment. Dot size indicates the number of genes in each KEGG pathway (Count), and dot color represents the −log_10_ (FDR). KEGG pathway enrichment analysis was performed using DAVID 2025 (v2025_1) with Mus musculus used as the reference background in DAVID. For upregulated genes, significance was defined as FDR < 0.05; for downregulated genes, a more permissive threshold of FDR < 0.10 with Count ≥ 3 was applied. KEGG, Kyoto Encyclopedia of Genes and Genomes; FDR, false discovery rate; DAVID, Database for Annotation, Visualization and Integrated Discovery.

Target genes of core phase-up miRNAs were predominantly enriched in pathways related to metabolic and energy regulation. These included insulin- and PI3K/Akt-related signaling pathways, together with broader energy-sensing and metabolic regulatory pathways. In addition, pathways associated with cellular adaptation and longevity regulation were enriched, along with stress- and hypoxia-related signaling and cellular senescence, suggesting an association between circadian phase and metabolic homeostasis as well as long-term cellular state (Figure 6a). Target genes of core exercise-up miRNAs also showed enrichment in pathways involved in metabolic and energy regulation, including insulin- and PI3K/Akt-related signaling and energy-sensing pathways. Additional enriched pathways were related to metabolic adaptation, stress responses, inflammatory signaling, and cellular remodeling processes such as autophagy and cellular senescence, indicating exercise-associated regulation of metabolic homeostasis and cellular adaptation (Figure 6a). For core phase-down miRNAs, enriched pathways were mainly associated with metabolic stress and redox-related processes. These included pathways linked to Advanced Glycation End Products–Receptor for Advanced Glycation End Products (AGE-RAGE) signaling in diabetic complications, porphyrin metabolism, and cofactor biosynthesis, together with regulatory signaling such as transforming growth factor beta (TGF-β) signaling, suggesting phase-dependent alterations in redox balance and metabolic stress responses (Figure 6b). Target genes of core exercise-down miRNAs were enriched in pathways related to metabolic stress, inflammation-associated signaling, and cellular regulation. These pathways included those associated with diabetic complications, innate immune signaling, and cellular senescence (Figure 6b).

Collectively, KEGG pathway enrichment analysis indicates that phase- and exercise-associated miRNAs are linked to distinct signaling pathway modules, with phase-related core miRNAs preferentially associated with metabolic and energy-regulatory pathways, and exercise-related core miRNAs more strongly associated with stress, inflammatory, and cellular adaptation pathways.

## 4. Discussion

### 4.1. Main Findings

Using a two-factor design combining circadian phase (ZT3 vs. ZT15) and long-term training (exe vs. sed) in HFD-induced obese mice, this study systematically characterized skeletal muscle-derived EV-enriched miRNA profiles and examined their potential functional relevance.

First, at the transcriptomic level, both circadian phase and exercise were associated with differences in EV miRNA composition. In the composite-effect analyses, the composite phase contrast identified six miRNAs, and the composite exercise contrast identified eight miRNAs meeting exploratory criteria. Among these, miR-1a-3p and miR-1b-5p appeared across both composite contrasts; however, this signal was primarily driven by the sedentary-phase comparison rather than an independent exercise effect, indicating a stable phase-associated signature with exercise introducing context-dependent modulation. Second, condition-specific comparisons further revealed context-dependent differences, as phase-associated miRNA differences varied between sedentary and exercise conditions, and exercise-associated effects differed by circadian phase. PCA corroborated these findings at the global level, demonstrating a stronger association of circadian phase with overall variance in EV miRNA expression than exercise. Third, intersection analyses based on miRTarBase-validated target genes showed that composite trends captured substantial components of the corresponding condition-specific patterns, while distinct subsets reflected phase- or exercise-specific modulation. Exploratory functional enrichment of core target genes revealed partly distinct biological themes, with phase-associated miRNAs preferentially linked to metabolic and iron–heme-related processes, and exercise-associated miRNAs linked to signaling, inflammatory, and transcription-related processes. These enrichment results are hypothesis-generating rather than evidence of definitive pathway activation.

### 4.2. Mechanistic Considerations and Biological Significance

#### 4.2.1. Circadian Regulation of EV Secretion

The numerically larger size metrics at ZT15 are consistent with circadian-phase-dependent remodeling of the EV-enriched secretome, plausibly reflecting rhythmic shifts in membrane lipid composition and cellular metabolic state. These descriptive shifts likely align with broader rhythmic programs, as circadian control governs over 40% of the transcriptome and coordinates muscle metabolism through peripheral clock genes [10,11]. EVs participate in circadian-related communication, and EV biogenesis is influenced by oscillations of core clock components [30]. Enhanced metabolic activity during the active phase is associated with differences in cellular metabolic state and membrane-related properties, aligning with prior evidence of time-dependent exercise effects on muscle oxidative metabolism [12,13]. Furthermore, the diurnal production of EVs is influenced by host feeding-entrained signals, including gut microbiota-derived EVs exhibiting diurnal regulation and activating hepatic gluconeogenesis [31]. This rhythmic regulation of lipid metabolism, encompassing fatty acid synthesis, β-oxidation, and associated redox metabolism [9], may further modulate the lipid composition and biophysical properties of the secreted vesicle membrane.

#### 4.2.2. Myogenic miRNAs Under Circadian and Training Control

The detection of miR-1a-3p and miR-1b-5p across composite contrasts, primarily driven by the sedentary-phase comparison, aligns with their known roles in myogenesis [32,33], suggesting a phase-associated EV miRNA signature in EV-enriched fractions. Several miRNAs, including miR-181d and miR-191, have been identified as circadian-rhythmic miRNAs co-expressed with core clock genes in mouse liver [14]. Acute-exercise studies report rapid increases in circulating EVs carrying myogenic miRNAs supporting a muscle–liver communication axis [3,4]. The miR-1 family upregulation in the composite phase contrast was primarily driven by the sedentary-phase comparison (ZT15-sed vs. ZT3-sed); this phase-associated signal was absent in the corresponding exercise-condition comparison (ZT15-exe vs. ZT3-exe), suggesting that chronic training may have attenuated the phase-related differential in miR-1 cargo between ZT15 and ZT3. More broadly, unlike the transient miRNA mobilization observed in acute exercise paradigms [3,4], the present 8-week intervention reflects long-term adaptive remodeling, which may account for the comparatively modest exercise-specific miRNA signal observed across contrasts. Downregulation of miR-122-5p at ZT15 reflects altered EV-associated miRNA composition; given the established metabolic relevance of exosomal miR-122 [34], its directional regulation and source attribution would benefit from dedicated tracing studies in future work. It should also be noted that miR-122 is canonically a hepatocyte-enriched miRNA [35,36]; its detection in ex vivo QUA-derived EV-enriched preparations therefore suggests that it reflects contributions from non-myofiber cell populations within the tissue or from circulating EV contamination during the ex vivo culture, rather than myofiber-intrinsic regulation.

Beyond the miR-1 family, the concurrent upregulation of miR-29a-3p, miR-29b-3p, and miR-29c-3p represented the most numerically prominent cluster in the sedentary-phase comparison. The miR-29 family has been associated with impaired insulin signaling, with elevated expression in insulin-responsive tissues under insulin-resistant and type 2 diabetic conditions and suppression of insulin-activated Akt activity in adipocytes, although the precise mechanisms remain unclear [37,38]. In the HFD context, their phase-associated upregulation at ZT15 under sedentary conditions may reflect a time-of-day-dependent alteration in EV-enriched miRNA cargoes. Notably, miR-29b-3p was downregulated in the ZT15-exe vs. ZT15-sed comparison but showed no significant exercise-associated difference at ZT3, suggesting a potentially ZT15-specific modulation by chronic training. Given the metabolic relevance of the miR-29 family, this pattern may warrant further investigation in the context of active-phase exercise and insulin signaling under HFD conditions. Whether the miR-29 family and miR-1 family responses share a common phase-dependent regulatory mechanism remains to be determined.

#### 4.2.3. Exercise Modulation of EV-Mediated Crosstalk in HFD

HFD and obesity are associated with insulin resistance [5] and have been linked to alterations in EV-mediated interorgan communication [34,39]. Notably, HFD-associated adipose EVs deliver miR-27a to skeletal muscle, repressing peroxisome proliferator-activated receptor gamma (PPARγ) and inducing insulin resistance [40]. The present results indicate that exercise-associated differences in EV miRNA profiles, while detectable, were context-dependent. This pattern is compatible with differences in the enrichment of miRNA target genes associated with signaling pathways under HFD conditions, as indicated by the functional enrichment analyses. In prior in vivo work, exercise-induced muscle-derived EVs have been shown to transport glycolytic enzymes to bone and to mediate exercise-induced cognitive protection via miRNA transfer to the brain [41,42]. The exercise-associated differences in EV miRNA profiles observed here are therefore consistent with a long-term adaptive remodeling framework, in which repeated exercise bouts progressively reshape EV cargo composition rather than reflecting transient acute responses, lending biological plausibility to the present findings. Intersection analyses further showed that composite-effect target sets accounted for a substantial proportion of condition-specific signals, while ZT3- and ZT15-specific subsets pointed to phase-contingent exercise regulation beyond the core shared patterns.

#### 4.2.4. Time-Dependent Functional Networks from Enrichment Analyses

The GO/KEGG enrichments reveal complementary time-dependent networks: core phase-up targets were mainly enriched in metabolic and intracellular signaling processes, whereas core phase-down targets were dominated by iron–heme-related processes. Core exercise-up targets showed enrichment consistent with mechanical/metabolic signaling, while core exercise-down targets emphasized inflammation- and transcription-related processes. Both sets converged on nutrient/energy pathways (including insulin- and PI3K/Akt-related signaling) [9], which is notable as multiple miRNAs are known to modulate insulin sensitivity via this pathway [43]. The enrichment of oxidative stress-related processes in phase-associated targets is consistent with circadian regulation of mitochondrial redox homeostasis [44] and with altered redox homeostasis under HFD conditions [45]. The modulation of inflammatory signals (e.g., IL-1β regulation) aligns with the potential for long-term training to ameliorate HFD-induced chronic inflammation [46], consistent with evidence that miRNA-mediated mitochondrial dysfunction contributes to inflammation-related metabolic stress [47].

The prominence of porphyrin/heme-related terms in phase-associated enrichments is consistent with prior evidence of circadian regulation of iron metabolism. Hepcidin, a central regulator of systemic iron homeostasis, exhibits diurnal rhythmicity [48], and core clock components such as brain and muscle ARNT-like 1 (BMAL1) have been implicated in pathways related to heme metabolism in peripheral tissues [9]. Given the role of heme in electron transport chain complexes, alterations in heme availability may influence mitochondrial oxidative function and redox balance under HFD-induced metabolic stress [44]. Although direct evidence linking these processes to EV-mediated signaling is still emerging, this enrichment pattern highlights an underexplored aspect of chrono-metabolic regulation.

### 4.3. Integration with Prior Work and the Working Model

These findings align with the concept of time-dependent exercise adaptation, as supported by studies showing greater metabolic benefits during the active phase [12,13]. Indeed, exercise-induced miR-136-3p has been shown to modulate glucose uptake and myogenesis by targeting nardilysin convertase, highlighting the specific role of extracellular miRNAs in training adaptations [49]. Extending prior acute-exercise EV research [4], the present long-term paradigm suggests persistent associations in muscle EV-enriched miRNA profiles and supports circadian phase as a relatively greater contributor to variance. Under HFD, our results align with evidence that EV signaling contributes to metabolic dysregulation, while exercise introduces context-dependent modulation of time-associated EV communication [5,6,50]. Although prior studies have linked circadian regulation to inflammatory and apoptotic pathways [51], our exploratory functional enrichment analysis is consistent with this framework, suggesting links between muscle-derived EV miRNA target gene networks and pathways implicated in cardiometabolic disease [52].

Integrating these observations, we propose a working model wherein circadian phase associates with baseline muscle EV miRNA profiles, and exercise adds adaptive modulation. Pending validation through in vivo EV tracing and functional studies in recipient tissues, these patterns provide a testable framework for interorgan metabolic communication in obesity, with hypothesized involvement of phase-associated myogenic signals (e.g., miR-1 family) and exploratory enrichment in iron–heme and metabolic signaling pathways as functional indicators rather than evidence of disease-specific activation.

### 4.4. Limitations and Future Directions

Several limitations of the present study should be acknowledged. As polymer-based precipitation may co-isolate non-vesicular components, preparations are described as EV-enriched in accordance with MISEV guidelines, and findings reflect the miRNA landscape of the muscle-derived EV-enriched secretome rather than purified vesicles. The small sample size (*n* = 3 biological replicates per group) constrains statistical power; accordingly, the present findings are interpreted as exploratory and hypothesis-generating, pending confirmation in larger independent cohorts with quantitative reverse transcription polymerase chain reaction (qRT-PCR) validation of candidate miRNAs and assessment of inter-individual variability. This study was conducted in male C57BL/6J mice at a specific age; generalizability to other strains, ages, and female animals remains to be established, and these constraints limit the translational relevance of the present findings to broader populations. Additionally, as this study was specifically designed to characterize the joint effects of circadian phase and chronic exercise under HFD-induced obesity, a standard diet comparison group was not included. The phase-associated EV miRNA patterns identified here therefore serve as a foundation for future diet-comparative and mechanistic investigations, including functional studies in recipient tissues and higher-resolution temporal profiling under constant conditions, aimed at distinguishing endogenous circadian regulation from light-driven effects and determining the broader physiological relevance of circadian regulation of muscle-derived EV signaling.

## 5. Conclusions

In HFD-induced obese mice, circadian phase was the dominant contributor to global variance in muscle-derived EV-enriched miRNA profiles, exceeding the contribution of exercise, as reflected by the phase-associated separation along PC1 (33.47% of total variance). miR-1a-3p and miR-1b-5p were detected across composite contrasts; condition-specific analyses demonstrated that this signal was primarily phase-driven. Both miRNAs, together with the miR-29 family, were upregulated at ZT15 relative to ZT3 under sedentary conditions; their co-occurrence in the composite exercise contrast reflected this phase-driven signal rather than independent exercise effects. Exercise-associated differences were context-dependent: miR-150-5p and let-7i-5p were downregulated at ZT3, and miR-486b-3p was downregulated at ZT15, under exercise relative to sedentary conditions. Exploratory functional enrichment of experimentally validated targets suggested that phase-associated miRNAs preferentially mapped to metabolic and iron–heme pathways, whereas exercise-associated miRNAs were linked to signaling, inflammatory, and transcription-related networks.

Collectively, these findings indicate that circadian phase, rather than exercise, is the dominant contributor to global variance in muscle-derived EV-enriched miRNA profiles under obesogenic conditions, with exercise introducing context-dependent adaptive modulation. Pending functional validation in vivo, the temporal dimension of exercise prescription may represent an additional variable in strategies targeting EV-mediated metabolic communication in this context. This study provides a foundational basis for the chronobiological investigation of muscle-derived EV secretome dynamics and highlights temporal specificity as a key dimension in EV-mediated exercise physiology research.

## Data Availability

The raw small RNA sequencing data in this study have been deposited in the DNA Data Bank of Japan Sequence Read Archive (DDBJ SRA) and are accessible through BioProject accession number PRJDB40101. The data will be made publicly available upon publication of this article. All other relevant data are included within the article and its Supplementary Materials.

## Supplementary Materials

The following supporting information can be downloaded at: https://www.mdpi.com/article/10.3390/nu18071076/s1, Table S1. Differential expression statistics of detected miRNAs; Table S2. miRTarBase-validated target genes for Venn diagram validation; Table S3. Gene Ontology biological process (GO-BP) enrichment results; Table S4. KEGG pathway enrichment results.

## Abbreviations

The following abbreviations are used in this manuscript:

AGE-RAGE	Advanced Glycation End Products–Receptor for Advanced Glycation End Products
Akt	protein kinase B
ARRIVE	Animal Research: Reporting of In Vivo Experiments
BMAL1	brain and muscle ARNT-like 1
DAVID	Database for Annotation, Visualization and Integrated Discovery
DDBJ SRA	DNA Data Bank of Japan Sequence Read Archive
DMEM	Dulbecco’s Modified Eagle Medium
EV	extracellular vesicle
exe	Exercise
FC	fold change
FDR	false discovery rate
GO	Gene Ontology
GO-BP	Gene Ontology biological process
HFD	high-fat diet
IL-1β	interleukin-1 beta
KEGG	Kyoto Encyclopedia of Genes and Genomes
MAPK	mitogen-activated protein kinase
miRNA	microRNA
miRTarBase	miRNA Target Interaction Database
MISEV	Minimal Information for Studies of Extracellular Vesicles
mRNA	messenger RNA
NGS	next-generation sequencing
NTA	nanoparticle tracking analysis
PBS	phosphate-buffered saline
PC	principal component
PCA	principal component analysis
PCR	polymerase chain reaction
PI3K	phosphoinositide 3-kinase
PPARγ	peroxisome proliferator-activated receptor gamma
qRT-PCR	quantitative reverse transcription polymerase chain reaction
QUA	quadriceps muscle
RNA	ribonucleic acid
ROS	reactive oxygen species
sed	Sedentary
SEM	standard error of the mean
TGF-β	transforming growth factor beta
VO_2_max	maximal oxygen uptake
VST	variance-stabilizing transformation
ZT	Zeitgeber Time

## References

[1] Théry C., Witwer K.W., Aikawa E., Alcaraz M.J., Anderson J.D., Andriantsitohaina R., Antoniou A., Arab T., Archer F., Atkin-Smith G.K. (2018). Minimal Information for Studies of Extracellular Vesicles 2018 (MISEV2018): A Position Statement of the International Society for Extracellular Vesicles and Update of the MISEV2014 Guidelines. J. Extracell. Vesicles.

[2] Bensimhon D.R., Kraus W.E., Donahue M.P. (2006). Obesity and Physical Activity: A Review. Am. Heart J..

[3] Whitham M., Parker B.L., Friedrichsen M., Hingst J.R., Hjorth M., Hughes W.E., Egan C.L., Cron L., Watt K.I., Kuchel R.P. (2018). Extracellular Vesicles Provide a Means for Tissue Crosstalk during Exercise. Cell Metab..

[4] Frühbeis C., Helmig S., Tug S., Simon P., Krämer-Albers E. (2015). Physical Exercise Induces Rapid Release of Small Extracellular Vesicles into the Circulation. J. Extracell. Vesicles.

[5] Aswad H., Forterre A., Wiklander O.P.B., Vial G., Danty-Berger E., Jalabert A., Lamazière A., Meugnier E., Pesenti S., Ott C. (2014). Exosomes Participate in the Alteration of Muscle Homeostasis during Lipid-Induced Insulin Resistance in Mice. Diabetologia.

[6] Jalabert A., Vial G., Guay C., Wiklander O.P.B., Nordin J.Z., Aswad H., Forterre A., Meugnier E., Pesenti S., Regazzi R. (2016). Exosome-like Vesicles Released from Lipid-Induced Insulin-Resistant Muscles Modulate Gene Expression and Proliferation of Beta Recipient Cells in Mice. Diabetologia.

[7] Bartel D.P. (2018). Metazoan MicroRNAs. Cell.

[8] D’Souza R.F., Woodhead J.S.T., Zeng N., Blenkiron C., Merry T.L., Cameron-Smith D., Mitchell C.J. (2018). Circulatory Exosomal miRNA Following Intense Exercise Is Unrelated to Muscle and Plasma miRNA Abundances. Am. J. Physiol.-Endocrinol. Metab..

[9] Panda S. (2016). Circadian Physiology of Metabolism. Science.

[10] Zhang R., Lahens N.F., Ballance H.I., Hughes M.E., Hogenesch J.B. (2014). A Circadian Gene Expression Atlas in Mammals: Implications for Biology and Medicine. Proc. Natl. Acad. Sci. USA.

[11] Dyar K.A., Lutter D., Artati A., Ceglia N.J., Liu Y., Armenta D., Jastroch M., Schneider S., De Mateo S., Cervantes M. (2018). Atlas of Circadian Metabolism Reveals System-Wide Coordination and Communication between Clocks. Cell.

[12] Sato S., Basse A.L., Schönke M., Chen S., Samad M., Altıntaş A., Laker R.C., Dalbram E., Barrès R., Baldi P. (2019). Time of Exercise Specifies the Impact on Muscle Metabolic Pathways and Systemic Energy Homeostasis. Cell Metab..

[13] Ezagouri S., Zwighaft Z., Sobel J., Baillieul S., Doutreleau S., Ladeuix B., Golik M., Verges S., Asher G. (2019). Physiological and Molecular Dissection of Daily Variance in Exercise Capacity. Cell Metab..

[14] Na Y.-J., Sung J.H., Lee S.C., Lee Y.-J., Choi Y.J., Park W.-Y., Shin H.S., Kim J.H. (2009). Comprehensive Analysis of microRNA-mRNA Co-Expression in Circadian Rhythm. Exp. Mol. Med..

[15] Wang S., Zhang Z., Huang J., Tong Y., Wu C., Kobori H., Ma S., Suzuki K. (2025). Circadian Phase Determines Tissue-Specific Adaptations to Long-Term Exercise in Obese Mice. Nutrients.

[16] Lynch H.J., Jimerson D.C., Ozaki Y., Post R.M., Bunney W.E., Wurtman R.J. (1978). Entrainment of Rhythmic Melatonin Secretion in Man to a 12-Hour Phase Shift in the Light/Dark Cycle. Life Sci..

[17] Sudo A., Miki K. (1995). Circadian Rhythm of Catecholamine Excretion in Rats after Phase Shift of Light-Dark Cycle. Ind. Health.

[18] Xu Z., Qin Y., Lv B., Tian Z., Zhang B. (2022). Effects of Moderate-Intensity Continuous Training and High-Intensity Interval Training on Testicular Oxidative Stress, Apoptosis and m6A Methylation in Obese Male Mice. Antioxidants.

[19] Cho J., Johnson B.D., Watt K.D., Niven A.S., Yeo D., Kim C.-H. (2022). Exercise Training Attenuates Pulmonary Inflammation and Mitochondrial Dysfunction in a Mouse Model of High-Fat High-Carbohydrate-Induced NAFLD. BMC Med..

[20] Mytidou C., Koutsoulidou A., Katsioloudi A., Prokopi M., Kapnisis K., Michailidou K., Anayiotos A., Phylactou L.A. (2021). Muscle-derived Exosomes Encapsulate myomiRs and Are Involved in Local Skeletal Muscle Tissue Communication. FASEB J..

[21] Estrada A.L., Valenti Z.J., Hehn G., Amorese A.J., Williams N.S., Balestrieri N.P., Deighan C., Allen C.P., Spangenburg E.E., Kruh-Garcia N.A. (2022). Extracellular Vesicle Secretion Is Tissue-Dependent Ex Vivo and Skeletal Muscle Myofiber Extracellular Vesicles Reach the Circulation in Vivo. Am. J. Physiol.-Cell Physiol..

[22] Kozomara A., Birgaoanu M., Griffiths-Jones S. (2019). miRBase: From microRNA Sequences to Function. Nucleic Acids Res..

[23] Kawanishi N., Tominaga T., Suzuki K. (2023). Electrical Pulse Stimulation-Induced Muscle Contraction Alters the microRNA and mRNA Profiles of Circulating Extracellular Vesicles in Mice. Am. J. Physiol.-Regul. Integr. Comp. Physiol..

[24] Love M.I., Huber W., Anders S. (2014). Moderated Estimation of Fold Change and Dispersion for RNA-Seq Data with DESeq2. Genome Biol..

[25] Afgan E., Baker D., Batut B., van den Beek M., Bouvier D., Čech M., Chilton J., Clements D., Coraor N., Grüning B.A. (2018). The Galaxy Platform for Accessible, Reproducible and Collaborative Biomedical Analyses: 2018 Update. Nucleic Acids Res..

[26] Bardou P., Mariette J., Escudié F., Djemiel C., Klopp C. (2014). Jvenn: An Interactive Venn Diagram Viewer. BMC Bioinform..

[27] Huang D.W., Sherman B.T., Lempicki R.A. (2008). Bioinformatics Enrichment Tools: Paths toward the Comprehensive Functional Analysis of Large Gene Lists. Nucleic Acids Res..

[28] Kanehisa M., Goto S. (2000). KEGG: Kyoto Encyclopedia of Genes and Genomes. Nucleic Acids Res..

[29] Huang D.W., Sherman B.T., Lempicki R.A. (2009). Systematic and Integrative Analysis of Large Gene Lists Using DAVID Bioinformatics Resources. Nat. Protoc..

[30] Tao S.-C., Guo S.-C. (2018). Extracellular Vesicles: Potential Participants in Circadian Rhythm Synchronization. Int. J. Biol. Sci..

[31] Tan J., Taitz J.J., Ni D., Potier-Villette C., Pinget G., Pulpitel T., Stanley D., Nanan R., Macia L. (2025). Gut Microbiota-Derived Extracellular Vesicles Exhibit Diurnal Regulation and Activate Hepatic Gluconeogenesis. Mol. Metab..

[32] Chen J.-F., Tao Y., Li J., Deng Z., Yan Z., Xiao X., Wang D.-Z. (2010). microRNA-1 and microRNA-206 Regulate Skeletal Muscle Satellite Cell Proliferation and Differentiation by Repressing Pax7. J. Cell Biol..

[33] Bjorkman K.K., Buvoli M., Pugach E.K., Polmear M.M., Leinwand L.A. (2019). miR-1/206 Downregulates Splicing Factor Srsf9 to Promote C2C12 Differentiation. Skelet. Muscle.

[34] Castaño C., Kalko S., Novials A., Párrizas M. (2018). Obesity-Associated Exosomal miRNAs Modulate Glucose and Lipid Metabolism in Mice. Proc. Natl. Acad. Sci. USA.

[35] Willeit P., Skroblin P., Kiechl S., Fernández-Hernando C., Mayr M. (2016). Liver microRNAs: Potential Mediators and Biomarkers for Metabolic and Cardiovascular Disease?. Eur. Heart J..

[36] Oda S., Takeuchi M., Akai S., Shirai Y., Tsuneyama K., Yokoi T. (2018). miRNA in Rat Liver Sinusoidal Endothelial Cells and Hepatocytes and Application to Circulating Biomarkers That Discern Pathogenesis of Liver Injuries. Am. J. Pathol..

[37] He A., Zhu L., Gupta N., Chang Y., Fang F. (2007). Overexpression of Micro Ribonucleic Acid 29, Highly Up-Regulated in Diabetic Rats, Leads to Insulin Resistance in 3T3-L1 Adipocytes. Mol. Endocrinol..

[38] Williams M.D., Mitchell G.M. (2012). MicroRNAs in Insulin Resistance and Obesity. Exp. Diabetes Res..

[39] Thomou T., Mori M.A., Dreyfuss J.M., Konishi M., Sakaguchi M., Wolfrum C., Rao T.N., Winnay J.N., Garcia-Martin R., Grinspoon S.K. (2017). Adipose-Derived Circulating miRNAs Regulate Gene Expression in Other Tissues. Nature.

[40] Yu Y., Du H., Wei S., Feng L., Li J., Yao F., Zhang M., Hatch G.M., Chen L. (2018). Adipocyte-Derived Exosomal MiR-27a Induces Insulin Resistance in Skeletal Muscle Through Repression of PPARγ. Theranostics.

[41] Lin H., Yin L., Liu W., Li R., Jiang T., Yang M., Cao Y., Wang S., Yu Y., Chen C. (2025). Muscle-Derived Small Extracellular Vesicles Mediate Exercise-Induced Cognitive Protection in Chronic Cerebral Hypoperfusion. Adv. Sci..

[42] Ma S., Xing X., Huang H., Gao X., Xu X., Yang J., Liao C., Zhang X., Liu J., Tian W. (2023). Skeletal Muscle-Derived Extracellular Vesicles Transport Glycolytic Enzymes to Mediate Muscle-to-Bone Crosstalk. Cell Metab..

[43] Zhong F.-Y., Li J., Wang Y.-M., Chen Y., Song J., Yang Z., Zhang L., Tian T., Hu Y.-F., Qin Z.-Y. (2021). MicroRNA-506 Modulates Insulin Resistance in Human Adipocytes by Targeting S6K1 and Altering the IRS1/PI3K/AKT Insulin Signaling Pathway. J. Bioenerg. Biomembr..

[44] Cela O., Scrima R., Rosiello M., Pacelli C., Piccoli C., Tamma M., Agriesti F., Mazzoccoli G., Capitanio N. (2025). Circadian Clockwork Controls the Balance between Mitochondrial Turnover and Dynamics: What Is Life… without Time Marking?. Biochim. Biophys. Acta BBA Bioenerg..

[45] Ulasov A.V., Rosenkranz A.A., Georgiev G.P., Sobolev A.S. (2022). Nrf2/Keap1/ARE Signaling: Towards Specific Regulation. Life Sci..

[46] Liu S., Li H., Zhang Y., Song H., Fu L. (2023). Exercise Ameliorates Chronic Inflammatory Response Induced by High-Fat Diet via Sestrin2 in an Nrf2-Dependent Manner. Biochim. Biophys. Acta BBA Mol. Basis Dis..

[47] Krishnan P., Branco R.C.S., Weaver S.A., Chang G., Lee C.-C., Syed F., Evans-Molina C. (2024). miR-146a-5p Mediates Inflammation-Induced β Cell Mitochondrial Dysfunction and Apoptosis. J. Biol. Chem..

[48] Park W.-R., Choi B., Kim Y.-J., Kim Y.-H., Park M.-J., Kim D.-I., Choi H.-S., Kim D.-K. (2022). Melatonin Regulates Iron Homeostasis by Inducing Hepcidin Expression in Hepatocytes. Int. J. Mol. Sci..

[49] Katayama M., Caria E., Van Simaeys D., Sanz A.Y., Barrès R., Caidahl K., Wiklander O.P., El-Andaloussi S., Berggren P.-O., Zierath J.R. (2025). Exercise Training-Induced Extracellular miR-136-3p Modulates Glucose Uptake and Myogenesis through Targeting of NRDC in Human Skeletal Muscle. J. Sport Health Sci..

[50] Deng Z., Poliakov A., Hardy R.W., Clements R., Liu C., Liu Y., Wang J., Xiang X., Zhang S., Zhuang X. (2009). Adipose Tissue Exosome-Like Vesicles Mediate Activation of Macrophage-Induced Insulin Resistance. Diabetes.

[51] Schober A., Blay R.M., Saboor Maleki S., Zahedi F., Winklmaier A.E., Kakar M.Y., Baatsch I.M., Zhu M., Geißler C., Fusco A.E. (2021). MicroRNA-21 Controls Circadian Regulation of Apoptosis in Atherosclerotic Lesions. Circulation.

[52] Yang G., Yang W. (2025). Regulating the Expression of Exercise-Induced Micro-RNAs and Long Non-Coding RNAs: Implications for Controlling Cardiovascular Diseases and Heart Failure. Front. Mol. Biosci..

